# Paradoxical reduction of plasma lipids and atherosclerosis in mice with adenine-induced chronic kidney disease and hypercholesterolemia

**DOI:** 10.3389/fcvm.2023.1088015

**Published:** 2023-02-09

**Authors:** Mugdha V. Padalkar, Alexandra H. Tsivitis, Ylona Gelfman, Mariya Kasiyanyk, Neil Kaungumpillil, Danyang Ma, Michael Gao, Kelly A. Borges, Puneet Dhaliwal, Saud Nasruddin, Sruthi Saji, Hina Gilani, Eric J. Schram, Mohnish Singh, Maria M. Plummer, Olga V. Savinova

**Affiliations:** ^1^Department of Biomedical Sciences, New York Institute of Technology College of Osteopathic Medicine, Old Westbury, NY, United States; ^2^Department of Clinical Specialties, New York Institute of Technology College of Osteopathic Medicine, Old Westbury, NY, United States

**Keywords:** triglycerides, cholesterol, chronic kidney disease, mouse model, atherosclerosis

## Abstract

**Background:**

Atherosclerotic cardiovascular disease is prevalent among patients with chronic kidney disease (CKD). In this study, we initially aimed to test whether vascular calcification associated with CKD can worsen atherosclerosis. However, a paradoxical finding emerged from attempting to test this hypothesis in a mouse model of adenine-induced CKD.

**Methods:**

We combined adenine-induced CKD and diet-induced atherosclerosis in mice with a mutation in the low-density lipoprotein receptor gene. In the first study, mice were co-treated with 0.2% adenine in a western diet for 8 weeks to induce CKD and atherosclerosis simultaneously. In the second study, mice were pre-treated with adenine in a regular diet for 8 weeks, followed by a western diet for another 8 weeks.

**Results:**

Co-treatment with adenine and a western diet resulted in a reduction of plasma triglycerides and cholesterol, liver lipid contents, and atherosclerosis in co-treated mice when compared with the western-only group, despite a fully penetrant CKD phenotype developed in response to adenine. In the two-step model, renal tubulointerstitial damage and polyuria persisted after the discontinuation of adenine in the adenine-pre-treated mice. The mice, however, had similar plasma triglycerides, cholesterol, liver lipid contents, and aortic root atherosclerosis after being fed a western diet, irrespective of adenine pre-treatment. Unexpectedly, adenine pre-treated mice consumed twice the calories from the diet as those not pre-treated without showing an increase in body weight.

**Conclusion:**

The adenine-induced CKD model does not recapitulate accelerated atherosclerosis, limiting its use in pre-clinical studies. The results indicate that excessive adenine intake impacts lipid metabolism.

## 1. Introduction

Chronic kidney disease (CKD) confers an increased risk of mortality from cardiovascular causes, including atherosclerotic cardiovascular disease (ASCVD) (1). CKD is considered a risk-enhancing factor for ASCVD (2, 3). Vascular calcification, another independent predictor of ASCVD used for risk stratification, is prevalent in patients with CKD (4, 5). It is currently unknown whether calcification directly contributes to an increased ASCVD burden or severity in patients with CKD (6). A better understanding of the complex interplay between calcification and atherosclerosis in patients with CKD could help develop new strategies to reduce ASCVD and mortality in these patients (6).

Animal models demonstrate that increased uremic toxins accelerate ASCVD (7). Uremic toxins induce endothelial dysfunction, inflammation, and oxidative stress, promoting atherosclerosis, while premature senescence and cell death contribute to plaque vulnerability (8–11). We hypothesized that a causal relationship exists between calcification and atherosclerosis in CKD and sought to test this hypothesis in an animal model.

Animal models of CKD and atherosclerosis almost exclusively use a 5/6 kidney ablation in mice deficient in the apolipoprotein E (*apoE*) or low-density lipoprotein receptor (*ldlr*) (12–15). The 5/6 nephrectomy model, however, has several limitations, including variable surgical success rate, inter-laboratory variations, and irreversibility of the procedure (16). On the other hand, several recent vascular calcification studies not concerned with atherosclerosis have been conducted using a non-surgical adenine-induced model of CKD (17–20). Excessive oral intake of adenine leads to the accumulation of 2,8-dihydroxyadenine crystals in the renal tubules that cause progressive tubulointerstitial nephropathy (21). Adenine-induced CKD is thought to be reversible, with kidney function fully or partially restored upon discontinuation of adenine treatment (22). The reversibility of the adenine model of CKD might provide an opportunity to study the effects of calcification on the progression of atherosclerosis independently of uremic blood toxins.

In this study, we explored two scenarios. The first experiment involved a concurrent induction of CKD and atherosclerosis by feeding *ldlr* mutant mice with a western diet supplemented with adenine to confirm the interaction between CKD and atherosclerosis. In the second study, CKD was established prior to atherosclerosis. Adenine was used as a pre-treatment and discontinued during a western diet treatment. Unexpectedly, we observed a striking reduction of plasma lipids and atherosclerosis in mice co-treated with adenine and a western diet despite all other manifestations of CKD. The development of atherosclerosis was no longer impeded after the discontinuation of adenine. Although the effect of adenine on lipid metabolism precluded testing of the central hypothesis of this study, the results point to a previously unrecognized mechanism of modulating atherosclerosis.

## 2. Materials and methods

### 2.1. Animal studies

Animal studies were approved by the Institutional Animal Care and Use Committee (IACUC) of the New York Institute of Technology College of Osteopathic Medicine (Protocol number 2020-OS-01) and complied with the National Institutes of Health Office of Laboratory Animal Welfare guidelines.

*Ldlr* mutant mice were initially obtained from the Jackson Laboratory (C57BL/6J-Ldlr^Hlb301^/J; strain #005061; Bar Harbor, ME, USA) in 2015 and kept in our colony. These mice carry a familial hypercholesterolemia mutation (C699Y) in the *ldlr* gene. At the time of discovery, the mutation was nicknamed “wicked high cholesterol” (*WHC*) (23). We used *WHC* mice in our earlier studies and reported their lipid profiles, atherosclerosis, and vascular calcification phenotypes (24, 25).

Mice were kept under a 12:12-h light/dark cycle with unrestricted access to food and water. A control diet (LabDiet 5001) was obtained from W.F Fisher and Son Inc. (Somerville, NJ, USA); a western diet (TD.88137) was obtained from Envigo (Madison, WI, USA). CKD was induced by adenine (Sigma, cat. #A8626; St. Louis, MO, USA) supplementation in a western diet (experiment 1) or a control diet (experiment 2).

In the first experiment, 24 10-week-old mice (50% males) were divided into two groups. One group was fed a western diet alone (W) for 8 weeks; the other group was fed a western diet supplemented with 0.2% adenine (W + A) for 8 weeks. In the second experiment, 82 10-week-old mice (50% males) were divided into four groups. The first group was fed a control diet for 8 weeks (C) and the second group was fed a control diet supplemented with adenine for 8 weeks (A). Due to high mortality in this experiment, the adenine dose for male mice was adjusted to 0.1%, whereas females received 0.2% adenine supplementation (26). Mice in the remaining two groups were fed a control diet with or without adenine for 8 weeks and then switched to a western diet without adenine supplementation for another 8 weeks (C→W and A→W).

### 2.2. Blood and urine chemistry

Blood was collected at terminal time points by cardiac puncture. Mice were fasted for 5 h prior to blood collection. Lithium heparin plasma was prepared and kept frozen until the analysis. Total cholesterol, triglycerides, and glucose were measured in whole plasma to evaluate lipid and carbohydrate metabolism. Blood urea nitrogen (BUN), a biomarker of kidney function, along with liver enzymes (indicative of hepatocellular death) alanine aminotransferase (ALT) and aspartate aminotransferase (AST) were measured after clearing the plasma with a Lipoclear reagent (Beckman Coulter, Inc., Brea, CA, USA) to reduce interference from lipemia. Urine samples were collected *via* metabolic cages (Techniplast, West Chester, PA, USA) to calculate 24-h glucose and protein excretion. All reagents were from Pointe Scientific (Lincoln Park, MI, USA) and used according to the manufacturer’s instructions. A mouse/rat Cystatin C Quantikine ELISA kit was obtained from R&D Systems (Minneapolis, MN, USA) as another measure of kidney function. Plasma IL-6 and TNF-α were measured using LEGEND MAX™ Mouse IL-6 and TNF-α ELISA kits (San Diego, CA, USA) as biomarkers of systemic inflammation.

### 2.3. Fecal fat content

Fresh fecal samples were collected after placing a mouse in a clean cage for 15–20 min. Fecal dry weight was determined following an overnight incubation at 42°C, and the samples were rehydrated with an equal volume (v/w) of saline. Lipids were extracted according to the Folch method and reconstituted in 0.1% Triton X-100 in saline. Cholesterol and free (non-esterified) fatty acids (FFA) were determined using Pointe Scientific cholesterol reagents and WAKO non-esterified fatty acids (NEFA) kit (FUJIFILM Medical Systems U.S.A. Inc., Lexington, MA, USA).

### 2.4. Histology

Tissues were harvested *via* whole-body perfusion and stored in 10% neutral formalin (Sigma, cat. #HT501320; St. Louis, MO, USA). The left kidneys were dissected, and transverse slices were made at the level of the renal pelvis. Cross-sectional slices of the liver (perpendicular to the central vein) were obtained from the medial lobes. Grossed tissues were equilibrated in 30% sucrose in phosphate-buffered saline (PBS), embedded in an Optimal Cutting Temperature (OCT) compound (Sakura Finetek USA, Inc., Torrance, CA, USA), and frozen in liquid nitrogen vapor. Ten μm-thick cryosections were prepared using a cryostat and mounted on positively-charged slides. Slides were stained with hematoxylin and eosin (H&E), picrosirius red (kidneys), and Oil red O (liver). Four high-power microscopic fields were imaged for each sample using an Olympus BX53 microscope (Olympus Co., Breinigsville, PA, USA). Kidney damage was assessed on a semi-quantitative pathological scale (0, 1, 2, 3) in H&E stained slides: 0 = no visible lesions; 1 = mild dilation of some tubules, luminal debris (casts), and partial nuclear loss in <1/3 tubules in a high-power field; 2 = apparent dilation of many tubules, nuclear loss, and cast in <2/3 tubules in a high-power field; 3 = severe dilation of most tubules, casts, and interstitial proliferation in >2/3 of the cortex (27). Kidney fibrosis was estimated as an average Picrosirius red-positive area. Liver damage was graded by analyzing hepatocellular vesicular steatosis on a scale (0, 1, 2, 3) from 0 = no damage to 3 > 75% affected area (28). Liver lipid content was measured as an Oil red O positive percent area. Interscapular brown adipose tissue (iBAT) was dissected. Ten μm-thick cryosections were prepared and mounted on TruBond360 adhesion slides and stained with H&E and Oil red O. Atherosclerosis was assessed at the aortic root level. Cryosections of the aortic roots were prepared over the as per recommended protocol (29). We collected 72 consecutive 10 μm sections as an array on eight slides. Slides were stained with Oil red O and Alizarin red. Atherosclerosis in the aortic root was quantified by measuring the Oil red O-positive area using ImageJ software Similarly, calcification was quantified by measuring the alizarin red positive area in sections of the aortic root.

### 2.5. Statistical analysis

Data were analyzed in GraphPad Prism 9 statistical software (San Diego, CA, USA). A two-way ANOVA was used to determine the main effects and the interactions between sex and adenine treatment (experiment 1) or adenine pre-treatment and a western diet (experiment 2). Not normally distributed non-zero data were log-transformed to meet the assumptions of the two-way ANOVA. *Post hoc* pair-wise comparisons were calculated using Sidak’s multiple comparison test. In addition, a *t*-test or a Mann–Whitney test were applied to investigate specific effects. Survival curves were compared by a log-rank (Mantel–Cox) test. Data were reported as means and standard deviations (SD). A *p*-value of <0.05 was considered statistically significant.

## 3. Results

### 3.1. Tubulointerstitial disease in *WHC* mice co-treated with adenine and a western diet

**FIGURE 1:**
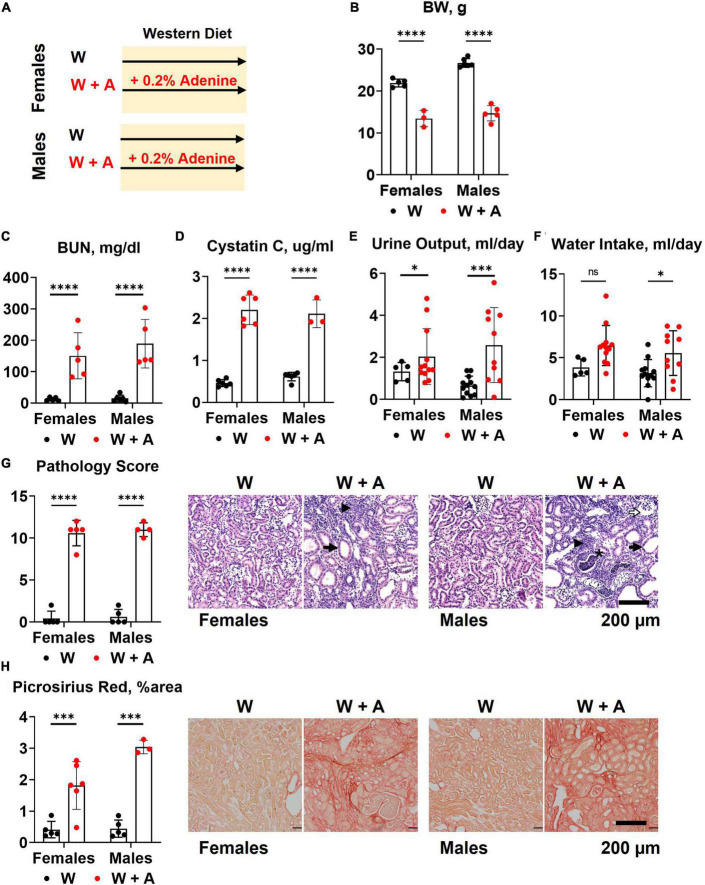
Chronic kidney disease in *WHC* mice fed a western diet supplemented with 0.2% adenine. **(A)** Treatment groups: male and female mice (*n* = 6 per group) were treated with a western diet (W) or a western diet supplemented with 0.2% adenine (W + A). **(B)** Body weight (BW). **(C)** Blood urea nitrogen (BUN). **(D)** Plasma Cystatin C. **(E)** 24-h urine output. **(F)** 24-h water intake. **(G)** Renal histology scores and representative images of H&E stained slides; tubular dilatation (arrow), inflammation (arrowhead), pus casts (asterisk), enlarged Bowman’s space (open arrow). **(H)** Quantification of kidney fibrosis and representative images of picrosirius red staining. *n* = 4–10 per group; two-way ANOVA; ns, not significant; *p < 0.05; ^***^p < 0.001; ^****^p < 0.0001.

Both male and female mice (*n* = 6 per group) were started on a western diet with or without 0.2% adenine supplementation when 10 weeks of age (Figure 1A). At the end of the 8-week treatment period, a significant loss of body weight (BW) was observed in both sexes (*p* < 0.0001, Figure 1B; Supplementary Table 1); one male mouse died. Adenine treatment was associated with a significant increase in plasma BUN and Cystatin C (*p* < 0.0001, two-way ANOVA; Figures 1C, D; Supplementary Table 2). A significant increase in 24-h water consumption and urine output was also observed in adenine-supplemented mice compared to those treated with a western diet alone (*p* < 0.0001, two-way ANOVA; Figures 1E, F; Supplementary Table 1). After 8 weeks on a diet, H&E staining of the renal cortex displayed hallmark features of adenine-induced CKD, including tubular dilation, granulomatous inflammation, pus casts, and enlarged Bowman’s space resulting in a significantly higher pathology score (*p* < 0.0001, two-way ANOVA; Figure 1G; Supplementary Table 1). The area positive for picrosirius red staining was also increased, indicating the development of interstitial fibrosis (*p* < 0.0001, two-way ANOVA; Figure 1H; Supplementary Table 1). Overall, these data confirmed the expected renal phenotype in mice treated with 0.2% adenine on a western diet (30).

### 3.2. Aortic root atherosclerosis, plasma lipids, and inflammation in *WHC* mice co-treated with adenine and a western diet

**FIGURE 2:**
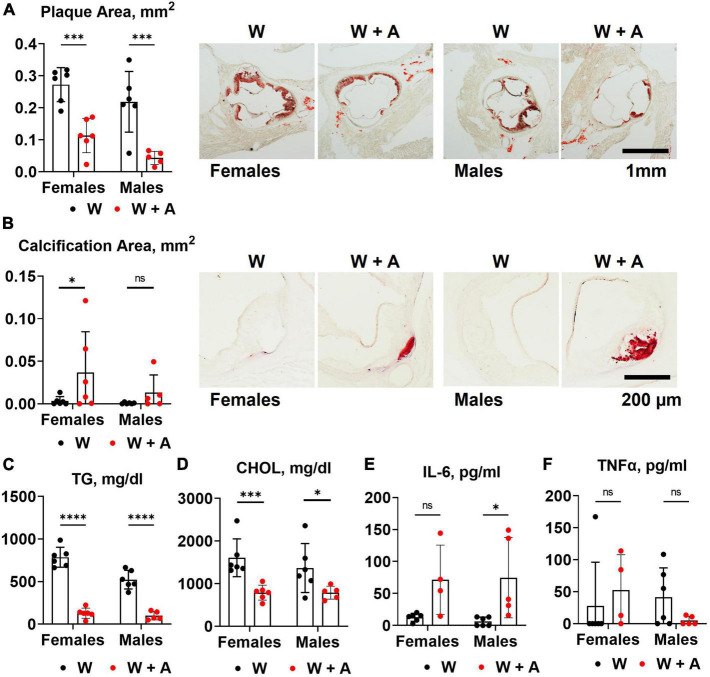
Aortic root atherosclerosis, plasma lipids, and inflammatory cytokines in *WHC* mice treated with a western with or without adenine. **(A)** Representative images and quantification of Oil red O staining of the aortic root. **(B)** Representative images and quantification of alizarin red staining of the aortic root. **(C)** Plasma triglycerides (TG). **(D)** Cholesterol (CHOL). **(E)** Interleukin-6 (IL-6). **(F)** Tumor necrosis factor alpha (TNFα). *n* = 4–6 per group; two-way ANOVA; ns, not significant; *p < 0.05; ^***^p < 0.001; ^****^p < 0.0001.

Unexpectedly, we found that adenine pre-treatment had a lowering effect on the aortic root atherosclerosis (*p* < 0.0001, two-way ANOVA; Figure 2A; Supplementary Table 3), despite the significant increase in vascular calcification (*p* < 0.05, two-way ANOVA; Figure 2B; Supplementary Table 3). In addition, plasma triglycerides and cholesterol were significantly reduced in adenine-treated mice compared with those treated with a western diet only (*p* < 0.0001, two-way ANOVA, Figures 2C, D; Supplementary Table 2). Because it was reported that adenine might suppress inflammatory cytokines secretion (31) and, thus, explain a reduction in atherosclerosis, we tested plasma levels of interleukin 6 (IL-6) and tumor necrosis factor alpha (TNFα). Contrary to this potential explanation, we found an increase in IL-6 level in mice co-treated with adenine and a western diet (*p* < 0.01, two-way ANOVA; Figure 2E; Supplementary Table 2). No significant changes in TNF-α were observed, with several mice displaying TNF-α levels below the detection limit in all groups (Figure 2F; Supplementary Table 2).

### 3.3. Food consumption and macronutrient excretion in feces and urine of *WHC* mice co-treated with adenine and a western diet

**FIGURE 3:**
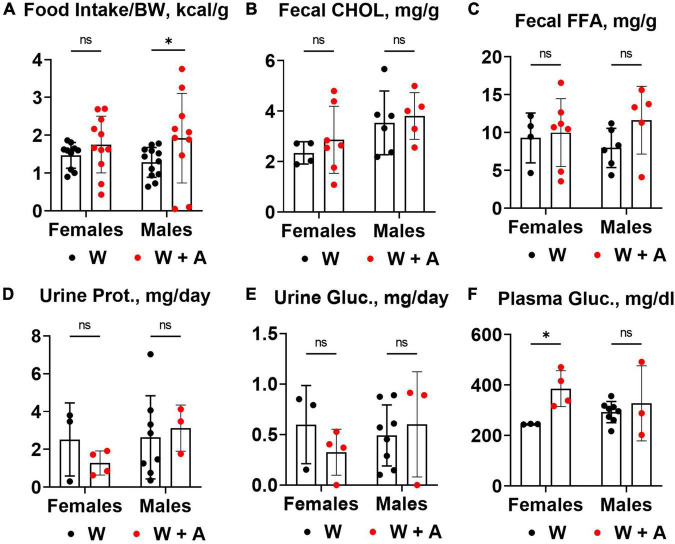
Food consumption and macronutrient excretion in feces and urine of WHC mice co-treated with adenine and a western diet. **(A)** Caloric intake per body weight (BW). **(B)** Fecal cholesterol (CHOL) content. **(C)** Fecal free fatty acids (FFA) content. **(D)** 24-h urine protein excretion. **(E)** 24-h urine glucose excretion. **(F)** Plasma glucose concentration. *n* = 3–11 per group; two-way ANOVA; ns, not significant; *p < 0.05.

We noted that mice treated with adenine had increased food consumption compared to mice from the western-only group (*p* < 0.05, two-way ANOVA; Figure 3A; Supplementary Table 4). There were no significant differences in fecal cholesterol or free fatty acids excretion in adenine treated mice compared to mice on a western diet alone (Figures 3B, C; Supplementary Table 4). No increase in urine protein or glucose excretion was found in adenine-treated mice compared to the western-only group (Figures 3D, E; Supplementary Table 4). An elevation in blood glucose was found in female mice co-treated with adenine and western; however, the overall effect was insignificant (Figure 3F; Supplementary Table 4).

### 3.4. Liver and brown adipose tissue phenotypes in *WHC* mice co-treated with adenine and a western diet

**FIGURE 4:**
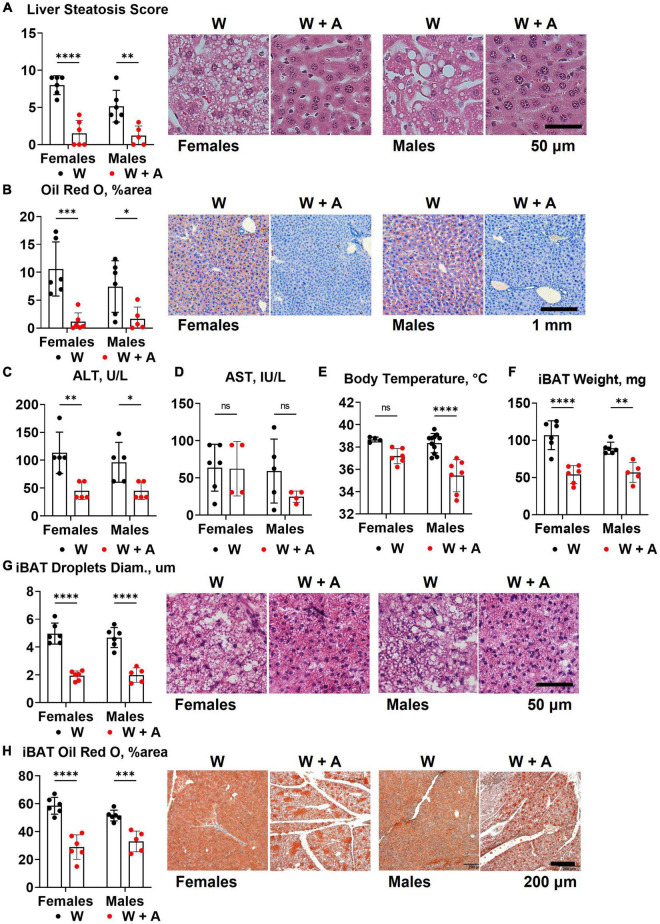
Liver and brown adipose tissue phenotypes of *WHC* mice treated with a western diet with or without adenine. **(A)** Pathology scores and representative images of H&E staining of the liver. **(B)** Representative images and quantification of Oil red O staining of the liver. **(C)** Plasma alanine transaminase (ALT). **(D)** Plasma aspartate transaminase (AST). **(E)** Core body temperature. **(F)** Interscapular brown adipose tissue (iBAT) weight. **(G)** Average diameter of lipid droplets in iBAT and representative images of iBAT stained with H&E. **(H)** iBAT lipis content detected by Oil red O staining. *n* = 4–11 per group; two-way ANOVA; ns, not significant; *p < 0.05; ^**^p < 0.01; ^***^p < 0.001; ^****^p < 0.0001.

The reduction of triglycerides and cholesterol in fasted plasma may result from liver toxicity and an imbalance in the secretion of triglyceride-rich, very low-density lipoproteins (VLDL) due to liver steatosis (32). Since liver steatosis is likely to develop in *WHC* mice fed a western diet for 8 weeks, we investigated whether adenine could have worsened steatosis. Contrary to this idea, H&E staining unequivocally showed that instead of worsening, adenine prevented steatosis (*p* < 0.0001, Figure 4A; Supplementary Table 5). Furthermore, analyzing Oil red O staining of the liver, we found very little accumulation of lipids in the liver of adenine-treated mice compared to the western-only group (*p* < 0.001, females; *p* < 0.05, males; Figure 4B; Supplementary Table 5). Conversely, no significant elevation of ALT or AST were detected in adenine-treated mice (Figures 4C, D); in fact, lower ALT levels were observed in adenine-treated mice compared to mice on a western diet alone (*p* < 0.001, two-way ANOVA; Figure 4C; Supplementary Table 2). Thus, the direct hepatotoxicity of adenine as a cause of lipid reduction was ruled out.

Reduction in body weight and inability to accumulate lipids in the liver in mice treated with adenine could indicate an increase in thermogenesis. To address this possibility, we measured core body temperature and interscapular brown adipose tissue (iBAT) mass and lipid content. We found that mice treated with adenine had lower body temperatures than mice from the western-only group (*p* < 0.0001, two-way ANOVA; Figure 4E; Supplementary Table 5). Adenine-treated animals also had smaller iBAT mass, the diameter of lipid droplets within iBAT tissue, and the overall lipid content of iBAT (*p* < 0.0001 for all, two-way ANOVA; Figures 4F–H; Supplementary Table 5). Thus, there was no evidence of increased thermogenesis of BAT hyperplasia in mice treated with adenine.

### 3.5. Two-step model of adenine-induced CKD and atherosclerosis

**FIGURE 5:**
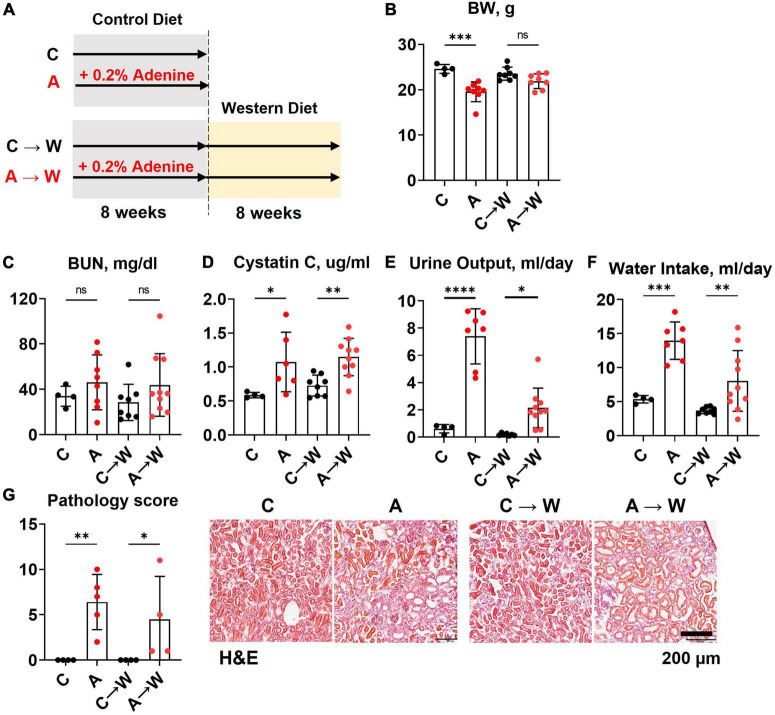
Chronic kidney disease in *WHC* female mice pre-treated with 0.2% adenine before a western diet. **(A)** Schematics of the experiment: control diet (C), 0.2% adenine in a control diet (A), control diet followed by a western diet (C→W), adenine diet followed by a western diet (A→W). **(B)** Body weight (BW). **(C)** Blood urea nitrogen (BUN). **(D)** Plasma Cystatin C. **(E)** 24-h urine output. **(F)** 24-h water intake. **(G)** Renal histology scores and representative images of H&E stained slides. *n* = 4–10 per group; two-way ANOVA. ns, not significant; *p < 0.05; ^**^p < 0.01; ^***^p < 0.001; ^****^p < 0.0001.

Unfortunately, the profound effect of adenine on lipid metabolism has precluded testing our central hypothesis regarding the interaction between vascular calcification and atherosclerosis in CKD. Although the lipid-lowering effect associated with adenine supplementation was serendipitous, it was interesting and potentially clinically relevant. Therefore, we proceeded with the second study to better understand the mechanism of the lipid-lowering effect of adenine (Figure 5A).

Both male and female mice were included in the study. However, despite a reduced adenine dose (0.1%), the mortality in male mice was prohibitively high (Supplementary Figure 1), and we could not complete the study in males. Therefore, the remaining data represent the findings in females only. Data were collected at the end of the adenine pre-treatment and after western diet treatment.

### 3.6. Kidney disease in *WHC* mice pre-treated with adenine before a western diet

Mice were treated with a control diet with or without adenine for 8 weeks and then switched to a western diet without adenine supplementation for another 8 weeks. At the end of the first 8-week treatment, a significant loss of body weight was observed in the adenine-supplemented mice compared to those on a regular diet (*p* < 0.001); the difference in body weight was not significant at the end of the western diet treatment (*p* = 0.0681, Figure 5B; Supplementary Table 6).

The BUN was not significantly affected by adenine in this experiment (Figure 5C; Supplementary Table 7). However, adenine pre-treated mice showed significant increases in plasma Cystatin C (*p* < 0.05), which persisted after discontinuation of adenine treatment (*p* < 0.01, Figure 5D; Supplementary Table 7). In addition, these mice also displayed significant polyuria and polydipsia immediately after the adenine treatment period and 8 weeks after they were switched to a western diet without adenine supplementation (Figures 5E, F; Supplementary Table 6).

H&E staining of the renal cortex revealed tubulointerstitial damage immediately after adenine pre-treatment (*p* < 0.01) that persisted until the end of the western diet treatment (Figure 5G; Supplementary Table 6).

### 3.7. Aortic root atherosclerosis and plasma lipids in *WHC* mice pre-treated with adenine before a western diet

**FIGURE 6:**
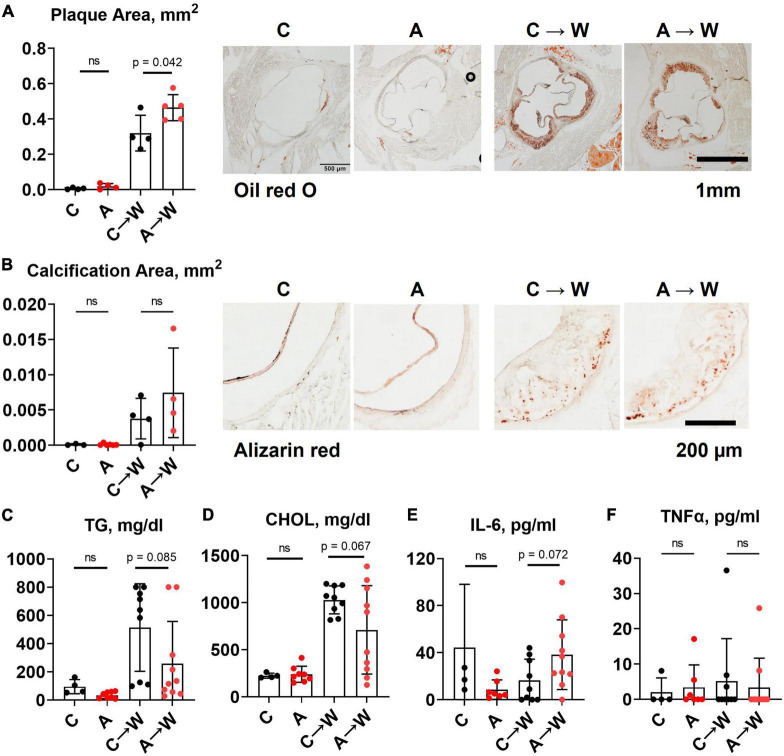
Aortic root atherosclerosis, plasma lipids, and inflammatory cytokines in *WHC* mice pre-treated with 0.2% adenine before a western diet. **(A)** Representative images and quantification of Oil red O staining of the aortic root. **(B)** Representative images and quantification of alizarin red staining of the aortic root. **(C)** Plasma triglycerides (TG). **(D)** Cholesterol (CHOL). **(E)** Interleukin-6 (IL-6). **(F)** Tumor necrosis factor alpha (TNFα). *n* = 4–6 per group; two-way ANOVA; ns, not significant.

Adenine pre-treatment did not affect aortic root plaque size (Figure 6A; Supplementary Table 8). However, a *t*-test comparison between adenine pre-treated and untreated mice detected a slight increase in plaque size in pre-treated mice compared to those not pre-treated (*p* = 0.0418). There was no significant difference in vascular calcification between the groups on the same diet (Figure 6B; Supplementary Table 8). Plasma triglycerides and cholesterol in adenine pre-treated mice were not significantly different compared to untreated mice on a western diet (Figures 6C, D; Supplementary Table 7). There were no differences in plasma inflammatory cytokines IL-6 and TNFα (Figures 6E, F; Supplementary Table 7) between adenine pre-treated and untreated mice on a western diet. This experiment demonstrated that the lipid-lowering effect of adenine in the setting of a western diet is reversible upon discontinuation of adenine and that adenine pre-treatment did not interfere with the development of aortic root atherosclerosis in *WHC* mice on a western diet.

### 3.8. Liver and brown adipose tissue phenotypes of *WHC* mice pre-treated with adenine before a western diet

**FIGURE 7:**
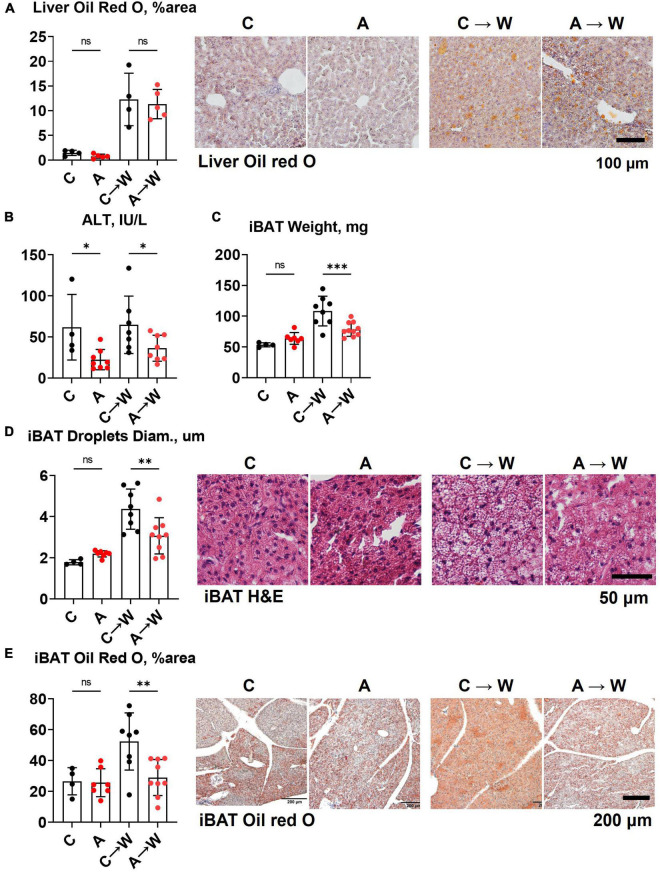
Liver and brown adipose tissue phenotypes in *WHC* mice pre-treated with adenine before a western diet. **(A)** Representative images and quantification of Oil red O staining of the liver. **(B)** Plasma alanine transaminase (ALT). **(C)** Interscapular brown adipose tissue (iBAT) weight. **(D)** Average diameter of lipid droplets in iBAT and representative images of iBAT stained with H&E. **(E)** iBAT lipis content detected by Oil red O staining. *n* = 4–9 per group; two-way ANOVA; ns, not significant; *p < 0.05; ^**^p < 0.01; ^***^p < 0.001.

In contrast to the first experiment involving the co-administration of adenine and a western diet, the liver lipid content quantified by Oil red O staining was similar between adenine pre-treated and untreated mice on a western diet (Figure 7A; Supplementary Table 9). Interestingly, ALT levels remained significantly lower in the adenine pre-treated mice compared to those untreated at both time points (Figure 7B; Supplementary Table 7). This experiment showed that the attenuation of hepatic steatosis by adenine appeared fully reversible upon its discontinuation. The size of interscapular brown adipose tissue (iBAT), lipid droplets diameter, and lipid content iBAT, however, was significantly reduced in adenine pre-treated mice 8 weeks after mice were switched to a western diet without adenine supplementation (Figures 7C–E; Supplementary Table 9).

### 3.9. Increased food intake and urinary macronutrient loss in *WHC* mice pre-treated with adenine before a western diet

**FIGURE 8:**
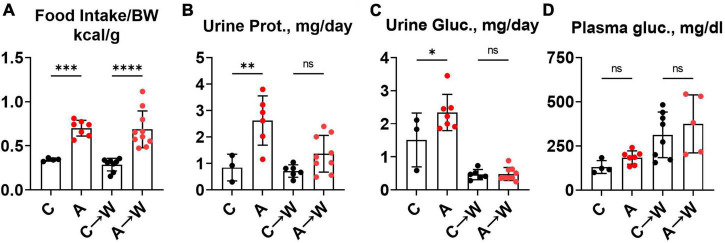
Food intake, urinary macromolecule loss, and plasma glucose in *WHC* mice pre-treated with adenine before a western diet. **(A)** 24-h calorie intake from food (control diet—LabDiets 5001; western—Envigo TD.88137). **(B)** 24-h urine protein excretion. **(C)** 24-h urine glucose excretion. **(D)** Fasted plasma glucose. *n* = 4–10 per group; two-way ANOVA; ns, not significant; *p < 0.05; ^**^p < 0.01; ^***^p < 0.001; ^****^p < 0.0001.

We noted in the first experiment that mice co-treated with adenine and a western diet consumed more calories from food than non-exposed mice (Supplementary Table 4). The increase in food consumption was also significant in mice pre-treated with adenine compared to those untreated, and this difference persisted after discontinuation of adenine (Figure 8A; Supplementary Table 10). This phenotype was striking because adenine-exposed mice did not gain weight (Figure 5B), suggesting that adenine exposure accelerated metabolism. A plausible explanation was that mice were losing calories due to their kidney disease. Indeed, mice had significant proteinuria and glucosuria while on the adenine treatment; however, both effects were not significant after the discontinuation of adenine (Figures 8B, C; Supplementary Table 10). Of note, although increased on a western, as expected (33), plasma glucose concentration was not affected by adenine treatment (Figure 8D; Supplementary Table 7).

### 3.10. Summary of findings

**FIGURE 9:**
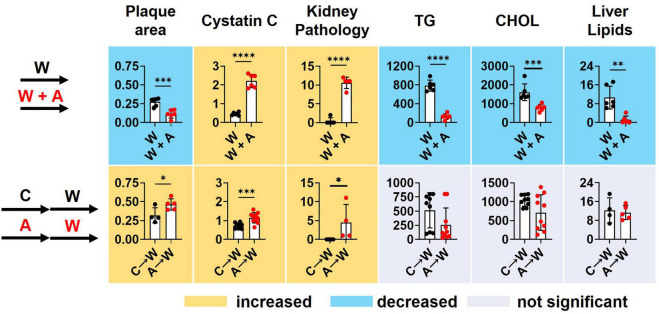
Summary of key findings. *T*-test; *p < 0.05; ^**^p < 0.01; ^***^p < 0.001; ^****^p < 0.0001.

Supplementing a western diet with 0.2% adenine for 8 weeks resulted in the development of CKD in *ldlr* mutant mice that was manifested as tubulointerstitial kidney damage and elevated plasma Cystatin C, a marker of kidney disease. In addition, adenine exposure suppressed hyperlipidemia and liver steatosis in *ldlr* mutant WHC mice on a western diet. Adenine-induced kidney disease persisted after its discontinuation. Plasma lipids and lipid metabolism in the liver appeared to rebound after discontinuation of adenine in *ldlr* mutant WHC mice consequently exposed to a western diet (Figure 9).

## 4. Discussion

The goal of this study was to model CKD in the background of hyperlipidemia using a non-surgical approach and then investigate the long-term effects of CKD-induced vascular calcification on the course of atherosclerosis. However, we were unable to test this hypothesis because of the unanticipated effect of adenine on lipid metabolism that masked the effect of vascular calcification on the extent of atherosclerosis. Adenine supplemented diet were used to induce CKD without surgery as previously described (30).

In the first experiment with a western diet and adenine co-treatment, we documented the reduction of renal function and tubulointerstitial pathology in male and female mice as expected. Surprisingly we found reduced plasma triglycerides, cholesterol, and atherosclerotic plaque formation, the findings that were opposite to a phenotype in a model of surgical kidney mass reduction (33, 34). In addition, we found a striking reduction of lipids in the liver without any signs of liver toxicity of adenine. Our observation raised the possibility that adenine might affect lipid metabolism.

While our manuscript was in preparation, two studies reported on atherosclerosis in adenine-treated *apoE* knockout mice. One study showed an increase in plasma lipids without an increase in atherosclerosis, attributed by the authors to an overall reduction in inflammation (35). In our experiment, we observed an increase in plasma IL-6 and TNFα suggesting that the reduction of atherosclerosis was independent of inflammation in *ldlr* mutant WHC mice. In the other study, *apoE* knockout mice were treated with adenine in a western diet supplemented with sodium cholate. In this study, the authors observed no changes in plasma or liver lipids but reduced atherosclerosis, which was explained by the increased cholesterol and triglycerides excretion in feces and increased bile acid synthesis (36). We investigated the fecal excretion of cholesterol and free fatty acids in *ldlr* mutant WHC mice and found no evidence of the increased fecal fat in adenine-treated mice under the conditions of our experiment on a western diet without sodium cholate.

In our follow-up experiment, we administered adenine and western diets sequentially. We observed a tubulointerstitial injury, polydipsia, and polyuria in adenine pre-treated mice that persisted after 8 weeks of adenine washout. Plasma lipids were highly variable between individual mice within the adenine-pretreated mice, and the liver lipid content increased after mice were switched from an adenine-supplemented diet to a western diet. Moreover, mice pre-treated with adenine had significantly increased food intake, consuming twice as many calories without significant weight gain or blood glucose elevation.

Various metabolic effects could potentially explain the paradoxical reduction of atherosclerosis in the adenine-induced model of CKD. We can speculate that adenine-induced tubular damage results in macronutrient wasting and negative energy balance that leads to the reduction of synthesis of triglycerides and lipoproteins in the liver. As renal function improves following the withdrawal of adenine (as indicated by the resolution of proteinuria and glucosuria), the liver lipid metabolism rebounds and correlates with an increase in atherosclerosis in WHC mice after discontinuing adenine treatment. Such a mechanism suggests an indirect effect of renal tubular function of lipid metabolism and atherosclerosis. Alternatively, as discussed below, adenine might directly impact energy metabolism and triglyceride synthesis.

Several studies demonstrate the beneficial effects of adenine (31, 37–40). Adenine can act as an allosteric activator of a fuel-sensing enzyme AMP-activated protein kinase (AMPK), increasing cellular glucose metabolism (37); it can delay senescence of cultured cells (38), reduce inflammatory cytokines secretion and adhesion of monocytes to endothelial cells (31, 39), and improve wound healing (40). In addition, activation of AMPK has been shown to reduce hyperlipidemia and hepatic steatosis, plausibly explaining the liver and plasma lipid findings (41).

We observed that mice either co-treated or pre-treated with adenine neither exhibited histologic evidence of liver damage nor elevated ALT levels, suggesting that adenine was not toxic to the liver. Our results support findings from other studies showing a lack of adenine hepatotoxicity (42, 43). However, another study reported a hepatotoxic effect of adenine as demonstrated by elevated liver enzymes in response to inflammation (44). This is in contrast with the results of our study, in which increased systemic inflammation was not associated with elevated liver enzymes in adenine-treated animals.

Our study was not without limitations. Unfortunately, we could not test our hypothesis regarding the interaction between vascular calcification and atherosclerosis because of the unanticipated effects of adenine-induced CKD on lipid metabolism. Thus, the results of our study can only generate additional hypotheses. In the pre-treatment experiment, a more extended adenine clearance period and the assessment of the levels of adenine in plasma before administrating the western diet could have helped differentiate between the direct effects of adenine and tubulointerstitial injury on lipid metabolism or atherosclerosis. Alternatively, a different nephrotoxic agent acting on the proximal tubule, such as aminoglycosides (45), could be used to rule out the direct effects of adenine on metabolism. Lastly, the lack of direct measurements of energy expenditure and brown adipose tissue remains a significant limitation for the mechanistic understanding of the described lipid-lowering effect of adenine. Unfortunately, developing these timelines and experimental designs was outside the scope of this study.

Adenine-induced CKD has a metabolic effect on lipid and energy metabolism, and it may not be an appropriate model to study the effect of CKD on atherosclerosis in animals. Nevertheless, our findings can help design future hypothesis-driven research to understand the pathophysiology of increased metabolism and reduced atherosclerosis in adenine-treated mice.

## Data availability statement

The raw data supporting the conclusions of this article will be made available by the authors, without undue reservation.

## Ethics statement

The animal study was reviewed and approved by Institutional Animal Care and Use Committee (IACUC) of the New York Institute of Technology College of Osteopathic Medicine.

## Author contributions

MVP, AHT, MS, and OVS conceived and designed the research. MVP, AHT, YG, MK, NK, DM, MG, KAB, PD, SN, SS, HG, EJS, MS, and OVS performed the research. MVP, AHT, EJS, MS, MMP, and OVS analyzed and interpreted the results. MVP wrote the first draft. MVP, KAB, and OVS edited the manuscript.
