# Supplementary material for: Paradoxical reduction of plasma lipids and atherosclerosis in mice with adenine-induced chronic kidney disease and hypercholesterolemia
Source: Front Cardiovasc Med. 2023 Feb 9;10:1088015. doi: 10.3389/fcvm.2023.1088015 (PMC9947538; doi:10.3389/fcvm.2023.1088015)
Supplement: Supplementary file 1 [file Data_Sheet_1.pdf]

Paradoxical Reduction of Plasma Lipids and Atherosclerosis in Mice with Adenine-induced Chronic Kidney Disease and Hypercholesterolemia

Mugdha V. Padalkar[1], Alexandra H. Tsivitis[1], Ylona Gelfman[1], Mariya Kasiyanyk[1], Neil Kaungumpillil[1], Danyang Ma[1], Michael Gao[1], Kelly A. Borges[1], Puneet Dhaliwal[1], Saud Nasruddin[1], Sruthi Saji[1], Hina Gilani[1], Eric J. Schram[1], Mohnish Singh[1], Maria M. Plummer[2], Olga V. Savinova[1]*

[1] Department of Biomedical Sciences, New York Institute of Technology College of Osteopathic Medicine, Old Westbury, NY, USA

[2] Department of Clinical Specialties, New York Institute of Technology College of Osteopathic Medicine, Old Westbury, NY, USA

*** Correspondence:**
Olga V. Savinova
osavinov@nyit.edu

Supplementary Material

Supplementary Figure

Supplementary Tables 1-10

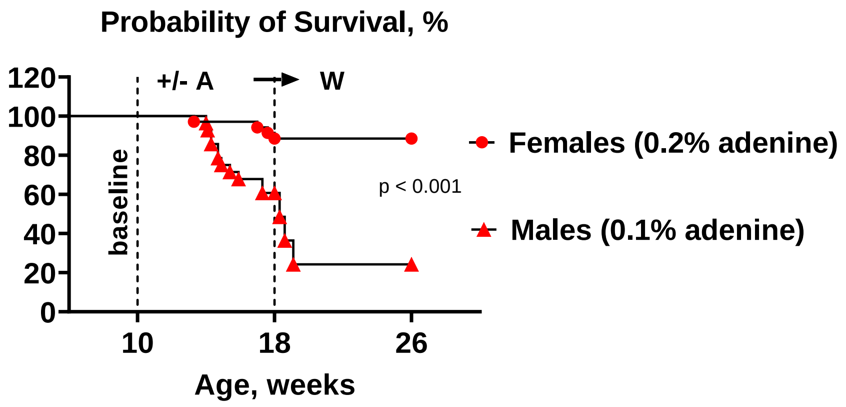

Supplementary Figure 1. Survival of female and male *WHC* mice pre-treated with adenine in a control diet followed by a western diet without adenine supplementation. Females were supplemented with 0.2% adenine; males were treated with a reduced dose of 0.1% adenine in a control diet. A log-rank Mantel-Cox test was used to compare the survival curves.

Supplementary Table 1. Body weight, water intake, urine output, kidney pathology: a two-way ANOVA analysis of the main effects and interaction between adenine treatment and sex

	Adenine		Sex		Interaction	
Dependent variable	% of total variation	p-value	% of total variation	p-value	% of total variation	p-value
BW, g	**87.67**	**<0.0001**	**5.682**	**0.0114**	1.248	0.1989
Water Intake, ml/day	**21.11**	**0.0025**	3.773	0.1775	0.0123	0.9377
Urine Output, ml/day	**21.14**	**<0.0001**	0.1131	0.8122	4.577	0.1367
Kidney Pathology Score	**96.30**	**<0.0001**	0.0817	0.5569	0.0091	0.8440
Log [Kidney Picrosirius Red, %area]	**75.68**	**<0.0001**	2.765	0.2109	2.097	0.2729

Log, log-transformed; BW, body weight

Supplementary Table 2. Blood chemistry: two-way ANOVA analysis of the main effects and interaction between adenine and sex in a western/adenine co-treatment study

	Adenine		Sex		Interaction	
Dependent variable	% of total variation	p-value	% of total variation	p-value	% of total variation	p-value
Log [BUN, mg/dL]	**87.43**	**<0.0001**	0.3322	0.5015	0.1232	0.6810
Cystatin C, μg/ml	**85.29**	**<0.0001**	0.0308	0.7762	0.4967	0.2618
TG, mg/dl	**82.06**	**<0.0001**	**6.005**	**0.0011**	**3.812**	**0.0063**
Log [CHOL, mg/dl]	**54.21**	**<0.0001**	1.588	0.4075	1.71	0.3905
IL-6, pg/ml	**44.76**	**0.0016**	0.0289	0.9253	0.2757	0.7725
TNFa, pg/ml	0.3632	0.7939	3.039	0.4532	9.897	0.1839
ALT, IU/ml	**57.35**	**0.0002**	1.17	0.5053	1.17	0.5053
AST, IU/ml	7.07	0.2645	9.796	0.1925	6.065	0.3
Plasma Glucose, mg/dl	**26.72**	**0.0308**	0.0926	0.8896	9.744	0.1690

Log, log-transformed; BUN, blood urea nitrogen; TG, triglycerides; CHOL, cholesterol; IL-6, interleukin 6; TNFa, tumor necrosis factor alpha; ALT, alanine transaminase; AST, aspartate transaminase;

Supplementary Table 3. Aortic root histology: two-way ANOVA analysis of the main effects and interaction between adenine treatment and sex in the adenine co-treatment study

	Adenine		Sex		Interaction	
Dependent variable	% of total variation	p-value	% of total variation	p-value	% of total variation	p-value
Plaque Area, mm^2	**63.48**	**<0.0001**	**8.684**	**0.0298**	0.1501	0.7608
Log [Calcification Area, mm^2]	**23.07**	**0.0481**	0.5341	0.7466	2.773	0.4652

Log, log-transformed

Supplementary Table 4. Food intake, and fecal and urine macronutrients: a two-way ANOVA analysis of the main effects and interaction between adenine treatment and sex in the adenine co-treatment study

	Adenine		Sex		Interaction	
Dependent variable	% of total variation	p-value	% of total variation	p-value	% of total variation	p-value
Log [Food Intake/BW, kcal/g]	**9.642**	**0.0462**	0.0040	0.9667	1.398	0.4377
Fecal CHOL, mg/g	2.876	0.4271	**20.84**	**0.0421**	0.2891	0.7996
Fecal FFA, mg/g	8.271	0.2086	0.0442	0.9251	3.778	0.3897
Urine Protein, mg/day	1.047	0.6868	7.014	0.3048	5.449	0.3636
Log [Urine Glucose, mg/day]	5.732	0.3863	3.117	0.5198	8.692	0.2899

Log, log-transformed; CHOL, cholesterol; FFA, free fatty acids

Supplementary Table 5. Liver and brown adipose tissue phenotypes: a two-way ANOVA analysis of the main effects and interaction between adenine treatment and sex in the adenine co-treatment study

	Adenine		Sex		Interaction	
Dependent variable	% of total variation	p-value	% of total variation	p-value	% of total variation	p-value
Liver Pathology Score	**96.30**	**<0.0001**	0.0817	0.5569	0.0091	0.8440
Log [Liver Oil Red O, %area]	**53.44**	**<0.0001**	1.587	0.4022	3.109	0.2452
Body Temperature, °C	**45.02**	**<0.0001**	**10.06**	**0.0135**	4.705	0.0808
iBAT Weight, mg	**69.18**	**<0.0001**	2.064	0.2159	3.843	0.0968
Log [iBAT Lipid Droplets Size, μm]	**86.57**	**<0.0001**	0.0760	0.7406	0.1220	0.6750
iBAT Oil Red O, %area	**74.78**	**<0.0001**	0.2915	0.5996	3.922	0.0650

Log, log-transformed; iBAT, interscapular brown adipose tissue

Supplementary Table 6. Body weight, water consumption, urine output, and kidney pathology: a two-way ANOVA analysis of the main effects and interaction between adenine pre-treatment and a western diet

	Adenine		Western		Interaction	
Dependent variable	% of total variation	p-value	% of total variation	p-value	% of total variation	p-value
BW, g	**40.30**	**<0.0001**	1.646	0.3403	**11.13**	**0.0181**
Log [Water Intake, ml/day]	**39.52**	**<0.0001**	**17.50**	**0.0015**	1.531	0.3031
Log [Urine Output, ml/day]	**62.48**	**<0.0001**	**16.07**	**<0.0001**	0.2541	0.5160
Kidney Pathology Score	**51.48**	**0.0017**	1.564	0.5032	1.564	0.5032

BW, body weight; Log, log-transformed

Supplementary Table 7. Blood chemistry: a two-way ANOVA analysis of the main effects and interaction between adenine pre-treatment and a western diet

	Adenine		Western		Interaction	
Dependent variable	% of total variation	p-value	% of total variation	p-value	% of total variation	p-value
Log [BUN, mg/dl]	5.738	0.2082	1.850	0.4703	0.8849	0.6165
Log [Cystatin C, μg/ml]	**40.31**	**0.0001**	4.098	0.1773	0.3945	0.6702
Log [TG, mg/dl]	**13.99**	**0.0092**	**30.90**	**0.0003**	0.1847	0.7491
Log [CHOL, mg/dl]	3.275	0.1539	**50.61**	**<0.0001**	3.688	0.1312
Log [IL-6, pg/ml]	1.361	0.4891	5.588	0.1684	**26.43**	**0.0054**
Log [TNFα, pg/ml]	0.056	0.9596	8.645	0.5003	4.520	0.6220
Log [ALT, U/L]	**21.69**	**0.0080**	7.056	0.1122	4.783	0.1872
Plasma Glucose, mg/dl	4.281	0.2255	**44.89**	**0.0006**	0.0234	0.9273

Log, log-transformed; BUN, blood urea nitrogen; TG, triglycerides; CHOL, cholesterol; IL-6, interleukin 6; TNFα, tumor necrosis factor alpha; ALT, alanine transaminase

Supplementary Table 8. Aortic root histology: a two-way ANOVA analysis of the main effects and interaction between adenine pre-treatment and a western diet

	Adenine		Western		Interaction	
Dependent variable	% of total variation	p-value	% of total variation	p-value	% of total variation	p-value
Log [Plaque Area, mm^2]	1.823	0.2121	**82.87**	**<0.0001**	0.2175	0.6578
Log [Calcification Area, mm^2]	1.623	0.3777	**67.25**	**<0.0001**	1.743	0.3612

Log, log-transformed

Supplementary Table 9. Liver and brown adipose tissue phenotypes: a two-way ANOVA analysis of the main effects and interaction between adenine pre-treatment and a western diet

	Adenine		Sex		Interaction	
Dependent variable	% of total variation	p-value	% of total variation	p-value	% of total variation	p-value
Liver Oil Red O, %area	0.4608	0.5707	**79.61**	**<0.0001**	0.01065	0.9309
iBAT Weight, mg	3.551	0.1210	**46.48**	**<0.0001**	**15.38**	**0.0026**
iBAT Lipid Droplets Size, μm	3.246	0.1437	49.31	**<0.0001**	**12.03**	**0.0077**
iBAT Oil Red O, %area	**11.97**	**0.0297**	**17.19**	**0.0106**	**10.24**	**0.0428**

Log, log-transformed; iBAT, interscapular brown adipose tissue

Supplementary Table 10. Food intake, and urinary macronutrient loss: a two-way ANOVA analysis of the main effects and interaction between adenine pre-treatment and a western diet

	Adenine		Western		Interaction	
Dependent variable	% of total variation	p-value	% of total variation	p-value	% of total variation	p-value
Log[Food Intake/BW, kcal/g]	**0.6387**	**<0.0001**	1.761	0.1780	0.6967	0.3916
Log[Urine Protein, mg/day]	**37.42**	**0.0008**	6.837	0.1069	5.041	0.1626
Log[Urine Glucose, mg/day]	2.556	0.0991	**59.09**	**<0.0001**	2.370	0.1114

Log, log-transformed
